# Shedding Light on the Lifestyle and Participation of Portuguese Adolescents with Chronic Conditions—Data from the HBSC 2018 Study

**DOI:** 10.3390/children9111717

**Published:** 2022-11-09

**Authors:** Ana Cerqueira, Fábio Botelho Guedes, Tania Gaspar, Emmanuelle Godeau, Margarida Gaspar de Matos

**Affiliations:** 1 Institute of Environmental Health (ISAMB), Aventura Social, Faculty of Medicine, University of Lisbon (FMUL), 1649-028 Lisbon, Portugal; 2 Faculty of Human Kinetics, University of Lisbon/FMH-UL, 1499-002 Lisbon, Portugal; 3 Digital Human-Environment Interaction Labs (HEI-LAB), Lusófona University of Humanities and Technologies, 1749-024 Lisbon, Portugal; 4 French School of Public Health, EHESP, 35043 Rennes, France; 5 CERPOP, UMR 1295, Unité Mixte UMR INSERM, Université Toulouse III Paul Sabatier, Team SPHERE, 31000 Toulouse, France; 6 APPSYci, ISPA, University Institute, 1149-041 Lisbon, Portugal

**Keywords:** adolescents, chronic conditions, school participation, lifestyle, quality of life

## Abstract

Variables associated with lifestyle can constitute either risk or protective factors for the development and progression of chronic conditions (CC). This study intends to explore the differences between adolescents with and without CC and between adolescents whose school participation is affected/not affected by the existing CC with regard to variables related to lifestyle (i.e., sleep, physical activity, BMI, and leisure). In addition, it also intends to analyze the influence of these variables (i.e., CC and lifestyle) regarding the adolescents’ quality of life (QoL). This work is part of the Portuguese HBSC 2018 study. A total of 8215 adolescents participated (52.7% female), with an average age of 14.36 years (*SD* = 2.28). The results showed that the adolescents with CC and whose school attendance and participation are affected by their CC exhibit more sleep difficulties (i.e., they experience lower sleep quality and have a higher degree of sleepiness), higher BMI levels (i.e., higher values of overweight and obesity), less participation in leisure activities, and a lower perception of QoL. A higher perception of QoL is associated with school participation unaffected by the existing CC, sleeping well, a low level of sleepiness, a more frequent practice of physical activity, a lower BMI, and a greater involvement in leisure activities. Adolescent health and well-being are a prominent issue in terms of public policies, with behavior and lifestyle playing a significant role in this domain. This message needs to be reinforced in regard to families, educators, healthcare professionals, and public sector policies, particularly concerning students with CC.

## 1. Introduction

The concept of a chronic condition (CC) encompasses different types of health conditions, including diabetes, as well as cardiovascular, oncological, and respiratory diseases [1,2]). Chronic diseases or non-communicable diseases (NCD) are long-term health conditions—generally slowly progressive and non-communicable from person to person—that require lifelong management [3,4]. According to the data from the 2014 and 2018 Health Behavior in School-Aged Children study in Portugal, 17.8% (*n* = 5757) and 15.1% (*n* = 3827), respectively, of adolescents reported having a chronic health condition [5,6].

These health conditions are based on a combination of genetic, physiological, environmental, and behavioral factors (e.g., lifestyle) [7] which are likely to affect the quality of life (QoL) of individuals [2,8]. Some of the characteristics associated with the experience of a CC may be associated with restrictions/limitations regarding the participation in various daily activities present in the adolescents’ lives (e.g., school, leisure activities, activities with their peers, etc.) which consequently are reflected in their well-being and QoL [8,9,10].

There are risk factors for the development of a CC that can be modifiable (e.g., sedentary lifestyle) or non-modifiable (e.g., genetic factors). Lifestyle is related to these factors, particularly the first. For example, the practice of physical activity (PA) can constitute either a risk or a protective factor for the development and progression of chronic health conditions [11,12,13,14]. The practice of these activities has positive effects in terms of the physical and mental health of young people, as well as the population in general, contributing to their psychosocial well-being and QoL [15,16,17,18,19]. Including the practice of physical activity in the individuals’ lifestyle has been associated with the prevention of chronic diseases. In addition, when the existence of this type of health condition is already verified, physical activity also appears as a facilitating element in its management [11,19,20,21,22,23].

The literature indicates that adolescents with CC tend to have more difficulties in terms of sleep [24,25,26,27]. Several studies show a relationship between sleep and chronic conditions such as asthma [25,28], diabetes [27,29], or oncological diseases [30,31,32]. A poor quality of sleep during adolescence can have long-term repercussions regarding problems related to sleep [33] and can affect both physical and mental health [26,34,35,36,37]. It should also be noted that sleep difficulties can result in daytime sleepiness [25,38].

The involvement in leisure activities that are meaningful to adolescents (i.e., that are relevant and serve a purpose) is a factor likely to influence their health, well-being, and psychosocial development [39,40]. These activities not only allow for the acquisition of different skills and knowledge, but also positively influence interpersonal relationships, emotional state [41,42,43], QoL, and well-being [42,44,45,46]. Moreover, it is important to emphasize that the health condition and the possible associated limitations/difficulties are factors likely to affect the participation in leisure activities and, consequently, the well-being of individuals [39,47].

A study by Snelgrove [48] showed that leisure activities occupy a prominent place in terms of shaping the adolescent’s identity. Adolescents with CC may experience difficulties in this context, since the presence of their health conditions can have repercussions in the following aspects: (1) the idealization of the body image (conditioning the image that they perceive as ideal or expected for their age or gender and, consequently, their willingness/motivation to engage in leisure activities); (2) the feeling of belonging and integration with peers (which once again affects their participation in leisure activities); (3) reducing their ability to participate in day-to-day and leisure activities (which is reflected in their well-being). These constraints associated with the CC can affect the adolescents’ identity construction [48], as well as their QoL [8].

The literature also points to the existence of a relationship between the Body Mass Index (BMI) and several chronic health conditions, namely cardiovascular diseases [49,50], liver disease [51] asthma [52,53,54,55,56,57,58], and diabetes [51,59,60,61]. Thus, BMI is a factor likely to influence the adolescents’ QoL [62].

The concern regarding the topic of obesity and its associated comorbidities has increased over time, receiving considerable attention from the research and health sectors [63]. It is crucial to consider the risks associated with the behavioral, psychological, and environmental dimensions regarding obesity prevention [64]. The adolescents’ lifestyle is reflected in their BMI, and it is important to promote the adoption of protective health behaviors to combat the development of obesity-related problems [63].

Adolescence is a phase with distinct characteristics that are associated with various psychosocial changes and challenges, so it is crucial to explore the factors associated with a good QoL [30]. This is especially true regarding the most vulnerable groups, as is the case for adolescents with CC [65,66]. Therefore, considering that these adolescents represent a group with higher risk in terms of lifestyle factors and level of participation in the different life contexts, this study aims to add to previous research on the adolescents’ lifestyle, focusing on the school participation of students with CC, rather than just on whether or not they have a CC. It also intends to contribute to an increase in knowledge regarding lifestyle-related variables and their influence on the adolescents’ QoL.

In this way, considering the evidence regarding the impact of CC and lifestyle-related variables on the adolescents’ QoL, the present study intends to explore and analyze some factors related to the lifestyle of Portuguese adolescents, with and without CC (i.e., sleep, physical activity, BMI and leisure activities), and their influence with regard to QoL. To achieve this, we intend to analyze the differences between adolescents with and without CC and with CC that does/does not affect the school participation and lifestyle-related variables, further influencing the impact of CC and lifestyle-associated variables on the adolescents’ QoL.

## 2. Method

This work is part of the Health Behavior in School-Aged Children/HBSC study [6,67], carried out in collaboration with the World Health Organization (WHO). The HBSC aims to study the behavior of adolescents in their life contexts and the influence of these behaviors on health and well-being. The study is carried out every 4 years, respecting an international protocol. In Portugal, the study has been developed since 1998 [68].

The HBSC 2018 study in Portugal was approved by the Ethics Committee and the MIME (Monitoring of School Surveys). It includes students in the 6th, 8th, 10th, and 12th grades, from several school groups. All the students participated voluntarily, and the informed consent was obtained from parents or legal guardians. The survey responses were anonymous and were obtained online.

### 2.1. Participants

A group of 8215 adolescents participated in this study, 52.7% (*n* = 4327) female, aged between 10 and 22 years, with an average age of 14.36 years (*SD* = 2.28). A total of 18.6% (*n* = 500) of girls and 13.6% of boys (*n* = 308) reported to have a CC. The sample is proportionally distributed over the five Portuguese regions (North, Central, Lisbon and Tagus Valley, Alentejo, and Algarve).

### 2.2. Measures and Variables

Taking into account the objectives of the present work, the variables shown in Table 1 were considered.

### 2.3. Data Analysis

Data analysis was carried out using the Statistical Package for the Social Sciences (SPSS) software, version 25 for Windows. Descriptive statistics were performed for all variables (mean, standard deviation, and percentages). The chi-square test for independent variables was used to analyze the relationship between having/not having a CC and CC affecting/not affecting school attendance and participation and the following variables: sleep, physical activity, participation in leisure activities, and BMI. The BMI variable was used categorically in the comparison of means to allow a better understanding of the results, since it is a very specific/technical categorization. The t-test of independent samples was used to analyze the relationship between having/not having a CC and the CC affecting/not affecting school attendance and participation, the degree of sleepiness, and the QoL.

Finally, two linear regression models were developed using the “Enter” method (adjusted to age and gender), to analyze the association between QoL and the other variables under study. A significance level of *p* < 0.05 was retained.

## 3. Results

The participants are characterized in Table 2, along with an analysis of the differences regarding adolescents with and without CC (16.3% and 83.7%, respectively). There are statistically significant differences for most of the variables under study, except for physical activity. In this study, adolescents with CC report worse quality of sleep and a higher degree of sleepiness. They also exhibit a higher BMI and participate in less leisure activities. On the other hand, adolescents without CC have a higher perception of QoL.

Table 3 presents the bivariate analysis of the differences between adolescents with and without school attendance and participation affected by the CC (27.1% and 72.9%, respectively). Statistically significant differences were found for most of the variables under study, except for physical activity.

The results demonstrate that adolescents with CC that affects school attendance and participation manifest more sleep problems (more difficulties in sleeping well and a higher degree of sleepiness), as well as a higher BMI and less involvement in leisure activities compared to those whose CC does not affect their school attendance/participation. On the other hand, adolescents without school attendance and participation affected by the existing CC have a higher perception of QoL.

The multiple linear regression model shown in Table 4 intends to explore the impact of variables related to the CC and to the adolescents’ lifestyle regarding their QoL. The model includes the variables used in the bivariate analysis (Table 3), adjusted for sex and age, *F*() = 406,26; *p* ≤ 0.001, and has a variance of 40.9%.

According to this model, only the CC does not appear as an explanatory variable for the QoL. Therefore, QoL is positively associated with sleeping well, with the practice of physical activity, and with the participation in more leisure activities. Additionally, QoL has a negative relationship with the degree of sleepiness and with the BMI, which means that a higher perception of QoL is associated with a low level of sleepiness and a lower BMI.

The multiple linear regression model in Table 4 was replicated (Table 5), substituting the variable CC (with/without CC) for the variable CC that affects/does not affect school attendance and participation. The model was adjusted for sex and age, and the variables included explained 41.0% of the variance, *F*(8711) = 62,77; *p* ≤ 0.001.

The analysis of this second model shows that the physical activity and the BMI variables do not appear to be explanatory of the QoL. Thus, QoL is positively associated with sleeping well and with involvement in leisure activities. Furthermore, QoL has a negative relationship with the degree of sleepiness, which means that a higher perception of QoL is associated with a low level of sleepiness.

It is noteworthy that the variable substituted in this model (i.e., CC that affects school attendance and participation) is positively related to the QoL, which means that having a CC that does not affect school attendance and participation is associated with a better perception of QoL

## 4. Discussion

This study aimed to explore the differences between the adolescents with and without CC and with and without school attendance and participation affected by the existing CC, with regard to variables related to lifestyle (i.e., sleep, physical activity, BMI, and leisure activities). Furthermore, it was also intended to analyze the influence of these variables (i.e., CC, sleep, physical activity, BMI, and leisure activities) on the adolescents’ QoL.

The results of this study allowed us to verify that the adolescents whose school attendance and participation are affected by their CC are the ones that show the worst indicators with regard to lifestyle variables and QoL. This is also true for the adolescents with CC as compared to those without CC. Thus, adolescents with CC and with school attendance and participation affected by their CC have more difficulties in sleeping (i.e., they sleep worse and have a higher degree of sleepiness), higher levels of BMI (i.e., higher values of overweight and obesity), less involvement in leisure activities, and a lower perception of QoL.

The literature shows the existence of a relationship between the quality and duration of sleep and the existence of certain chronic conditions (e.g., asthma, diabetes, oncological diseases). In turn, this relationship is reflected in the QoL of these adolescents [25,27,28,29,30,31,32]. Furthermore, there is evidence of the existence of more sleep difficulties in the adolescents with CC [24,25,26,27]. Several studies reveal that these adolescents tend to have higher levels of sleepiness compared to their peers [38,71,72], which is line with the results observed in the present study.

The literature also reveals a relationship between BMI and CC in young people [49,50,51,52,53,54,55,56,57,58,59,60,61], In this way, obesity is not only a risk factor for the onset of a CC, but also for its aggravation [52,53,54,63]. The results of our study are in line with those obtained by Lampalo et al. [55] and by Lennerz et al. [62], which indicate that patients with asthma have higher levels of BMI, and that QoL decreases as a result of the increase in the adolescents’ degree of obesity.

The involvement in leisure activities can be a protective factor regarding the experience and management of a CC, and it is associated with the perception of QoL [73]. It is noteworthy that the absence of involvement in meaningful activities may reflect an increased probability of the emergence of risk behaviors or health-related problems [40]). If there is already an associated risk regarding the reduction in the adolescents’ participation in leisure activities (i.e., the presence of a CC), the importance of promoting this participation gains even greater significance [74]. In this way, the results of our study are in line with the literature, insofar as the experience of a CC represents a greater risk for these adolescents to experience difficulties in participating in leisure activities [48].

In addition to the existence of a CC, it is important to consider whether this health condition affects the adolescents’ participation in the different life contexts (e.g., school, leisure activities), since the literature shows that these restrictions are reflected in their psychosocial functioning and QoL [8,10]. The results obtained in our study show that a higher QoL is associated with school participation not being affected by the existing CC, sleeping well, a lower degree of sleepiness, a more frequent practice of physical activity, a lower BMI, and a greater involvement in leisure activities. Therefore, this study reflects that more than just having a CC, the fact that this health condition affects the students’ school participation has a greater impact on their QoL. This result can also be observed in a study by Cerqueira et al. [75] that focuses on school-related variables regarding students with CC. This illustrates the importance of promoting the participation in different life contexts (e.g., school, family, friends) of adolescents with CC.

The adolescents’ health and well-being are a prominent issue in terms of public policies, with sleep-related issues having a significant role in this domain [76]. The results obtained in relation to sleep (sleeping well and degree of sleepiness) are in line with those from other studies that show that the adolescents’ sleep behavior is a factor that can have an impact on their well-being [14,24,77]. A longer duration and quality of sleep is related to better health indicators in children and adolescents, including in terms of QoL and well-being [37,78]. The results of Ridner et al. [77] are similar to those obtained in this study, observing that sleep quality is a strong predictor of the adolescents’ well-being.

The literature shows the existence of several comorbidities associated with obesity, and these are also reflected in the adolescents’ well-being [62,63,64]. Thus, BMI is a factor that can influence the adolescents’ QoL [62], which is in line with the results of this study.

Adolescents’ involvement in meaningful activities is another relevant aspect for their QoL. It is noteworthy that the absence of this involvement may reflect an increased probability of the emergence of risk behaviors or health-related problems [40]. In the vein, a study by Badura et al. [79] revealed that the participation in leisure activities is associated with better physical and mental health. It is important to promote this participation at an early stage of development, not only because it is a health-promoting element, but also because the way in which children spend their spare time will be reflected in their adult behavior in this domain [74,80].

Regarding physical activity, the results of our study are in line with those of the literature in the sense that this practice is reflected in an increase in well-being and QoL [15,16,17,18,19]. In general, the literature suggests that young people with CC tend to have lower levels of academic, physical, and social functioning compared to their peers [81]. Thus, adolescents with CC may experience more difficulties or barriers in the practice of physical activity. However, the involvement in these activities brings benefits in terms of physical and mental health, as is also true for young people without these health conditions [82]. Therefore, the importance of physical activity in terms of prevention and management of a CC should be reinforced [11,19,20,21,22,23].

A study by Healy et al. [24] demonstrated that adolescents with CC tend to be at higher risk in terms of not complying with guidelines regarding health behaviors, namely those related to sleep, physical activity, and sedentary behavior. However, what the present study adds to the existing literature is not only the differences between adolescents with and without CC, but also the focus on the participation of adolescents with CC and the associated impact of this participation on their QoL.

This study had some limitations that must be acknowledge: the self-reporting character of the data can have some associated bias (e.g., memory bias); the results are from students attending the public-school system, which means that the students attending the private school system and the ones that are not attending an educational institution (for different reasons) are not included; moreover, the cross-sectional design of the study does not allow for temporal organization and causality outcomes. However, it should be stressed that the HBSC follows a rigorous methodology, it allows for comparisons between the different study waves and countries involved and contains a large representative sample of Portuguese adolescents.

## 5. Conclusions

Aspects related to the health and well-being of adolescents are fundamental in terms of public policies, in which behavior and lifestyle play a significant role. There are several factors that can contribute to the development of a chronic health condition or to the worsening of an existing condition. The results observed in this study reinforce the importance of promoting healthy lifestyles among groups at higher risk, such as the adolescents whose school participation is affected by a CC, as they face increased difficulties compared to their peers.

Since the experience of a CC adds even more challenges to the adolescents’ daily lives, it is essential to design interventions tailored to those who live with this type of health condition [83,84]. It is also crucial to invest in the development of health and education programs, as well as measures that focus on preventing these problems and promoting the adoption of health enhancing behaviors. Adolescents with CC cannot be left out, and it is important to encourage them to actively participate in the design and implementation of programs and measures to promote their positive development, health, and well-being. It is essential to listen to the adolescents regarding the identification of their needs and difficulties. It is also important to involve them in the search for and implementation of solutions. This will contribute to the increase in their participation in decisions and actions that are aimed at the adoption of healthy lifestyles and, consequently, at increasing their well-being. It is also crucial to create and reinforce responses that support adolescents with CC, as well as their families, in the stage of transition from pediatric to adult care. This is because it is fundamental to promote health literacy so that adolescents are able to adopt a protective and preventive behavior in relation to their health and well-being.

As previously mentioned, what actually causes a greater impact on the QoL is not having a CC itself, but the situation in which this health condition affects school participation. This finding reinforces the importance of promoting the participation of adolescents with CC in their different life contexts (e.g., school, family, friends) which, in turn, can contribute to healthier lifestyles. This is a key message that must be enforced in regard to families, educators, healthcare professionals, and public policies.

## Figures and Tables

**Table 1:** Measures and variables under study.

Variables	Measure
Gender	1—Male; 2—Female
Chronic condition (CC)	1—Yes; 2—No
CC affects school attendance and participation (only adolescents with CC)	1—Yes; 2—No
Sleep well	1—No; 2—Yes
Degree of sleepiness	Scale adapted from Cantril [69], consisting of 10 steps, where the lowest step (1) corresponds to a low level of sleepiness and the highest step (10) to a high level of sleepiness
Physical activity (PA)	1—PA ≤ 3 times a week; 2—PA ≥ 4 times a week
Body Mass Index (BMI)	The Body Mass Index was categorized following the criterion of Cole et al. [70]: 1—Thinness; 2—Normal weight; 3—Overweight; 4—Obesity
Participation in leisure activities	1—Never/Rarely; 2—Often/Always
Quality of life (QoL)	Scale with 10 items on a 5-point Likert scale (1—Never, and 5—Always), with a minimum score of 5 and a maximum score of 50. Higher values reveal a better perception of quality of life, *α* = 0.83

**Table 2:** Population characteristics and bivariate analysis of differences between adolescents with and without a CC.

	*M* ± *SD* or % (*n*)			*p*
	Total (*n* = 8215)	Chronic condition (Yes) 16.3% (*n* = 808)	Chronic condition (No) 83.7% (*n* = 4150)	
Sleep well ^2^ No Yes	5.4 (275) 94.6 (4777)	* 7.5 (60) 92.5(736)	4.8 (196) * 95.2 (3853)	≤0.01
Degree of sleepiness ^1^	4.28 ± 2.63	4.64 ± 2.53	4.20 ± 2.64	≤0.001
Physical activity (PA) ^2^ PA ≤ 3 times a week PA ≥ 4 times a week	58.5 (4662) 41.5 (3302)	63.6 (514) 36.4 (294)	62.6 (2596) 37.4 (1554)	0.569
Body Mass Index (BMI) ^2^ Thinnes Normal weight Overweight Obesity	10.8 (828) 70.7 (5416) 15.4 (1182) 3.1(237)	9.2 (73) 70.9 (561) 15.4 (122) * 4.4 (35)	10.3 (414) 72.3 (2904) 14.9 (598) 2.6 (103)	≤0.05
Participation in leisure activities ^2^ Never/Rarely Often/Always	20.1 (1000) 79.9 (3963)	* 24.8 (200) 75.2 (608)	19.3 (799) * 80.7 (3350)	≤0.001
Quality of life (QoL) ^1^	36.43 ± 7.28	35.20 ± 7.13	36.68 ± 7.28	≤0.001

^1^ Independent sample *t*-test; ^2^ Chi-square test. * Adjusted residuals > 1.96. Abbreviations: *M*, mean; *SD*, standard deviation.

**Table 3:** Bivariate analysis of differences between adolescents with and without school attendance and participation affected by the CC.

	*M* ± *SD* or % (*n*)		*p*
	CC affects school attendance and participation (Yes) 27.1% (*n* = 198)	CC affects school attendance and participation (No) 72.9% (*n* = 532)	
Sleep well ^2^ No Yes	* 11.7 (23) 88.3 (173)	5.8 (31) * 94.2 (500)	≤0.01
Degree of sleepiness ^1^	4.97 ± 2.59	4.51 ± 2.48	≤0.05
Physical activity (PA) ^2^ PA ≤ 3 times a week PA ≥ 4 times a week	66.7 (132) 33.3 (66)	62.4 (332) 37.6 (200)	0.400
Body Mass Index (BMI) ^2^ Thinnes Normal weight Overweight Obesity	7.7 (15) 64.4 (125) * 21.1 (41) 6.7 (13)	10.4 (54) * 72.7 (379) 12.9 (67) 4.0 (21)	≤0.01
Participation in leisure activities ^2^ Never/Rarely Often/Always	* 34.3 (68) 65.7 (130	20.7 (110) * 79.3 (422	≤0.001
Quality of life (QoL) ^1^	32.96 ± 7.99	36.09 ± 6.47	≤0.001

^1^ Independent sample *t*-test; ^2^ Chi-square. * Adjusted residuals > 1.96. Abbreviations: *M*, mean; *SD*, standard deviation.

**Table 4:** Linear regression model of variables for the study of the QoL—predictors: CC and variables associated with lifestyle (sleep, physical activity, BMI, and leisure).

	Non-Standardized Coefficient		Standardized Coefficient	*t*
	B	Standard Error	*β*	
Chronic condition (CC)	0.35	0.22	0.02	1.60
Sleep well	3.24	0.15	0.26 ***	22.32
Degree of sleepiness	−0.34	0.03	−0.12 ***	−10.59
Physical activity (PA)	1.02	0.17	0.07 ***	5.87
Body Mass Index (BMI)	−0.09	0.02	−0.04 ***	−3.56
Participation in leisure activities	7.97	0.21	0.44 ***	37.99

The results were adjusted for age and sex. The variables were entered using the “Enter” mode. *** *p* ≤ 0.001.

**Table 5:** Linear regression model of variables for the study of the QoL—predictors: CC affects/does not affect school participation and variables associated with lifestyle (sleep, physical activity, BMI, and leisure).

	Non-Standardized Coefficient		Standardized Coefficient	*t*
	B	Standard Error	*β*	
CC affects school attendance and participation	1.15	0.46	0.07 **	2.49
Sleep well	3.23	0.34	0.29 ***	9.61
Degree of sleepiness	−0.44	0.08	−0.16 ***	−5.26
Physical activity (PA)	0.25	0.43	0.02	0.58
Body Mass Index (BMI)	−0.07	0.05	−0.04	−1.18
Participation in leisure activities	6.81	0.49	0.42 ***	14.01

The results were adjusted for age and sex. The variables were entered using the “Enter” mode. ** *p* ≤ 0.01; *** *p* ≤ 0.001.

## Data Availability

The data that support the findings of this study are available from the corresponding author upon reasonable request.
